# Cyclodextrin as Functional Carrier in Development of Mucoadhesive Tablets Containing *Polygoni cuspidati* Extract with Potential for Dental Applications

**DOI:** 10.3390/pharmaceutics13111916

**Published:** 2021-11-12

**Authors:** Magdalena Paczkowska-Walendowska, Emilia Szymańska, Katarzyna Winnicka, Dominik Szwajgier, Ewa Baranowska-Wójcik, Marek A. Ruchała, Marek Simon, Judyta Cielecka-Piontek

**Affiliations:** 1 Department of Pharmacognosy, Poznan University of Medical Sciences, Święcickiego 4, 60-781 Poznan, Poland; jpiontek@ump.edu.pl; 2 Department of Pharmaceutical Technology, Medical University of Białystok, Mickiewicza 2c, 15-222 Białystok, Poland; emilia.szymanska@umb.edu.pl (E.S.); kwin@umb.edu.pl (K.W.); 3 Department of Biotechnology, Microbiology and Human Nutrition, University of Life Sciences in Lublin, Skromna 8, 20-704 Lublin, Poland; dominik.szwajgier@up.lublin.pl (D.S.); ewa.baranowska@up.lublin.pl (E.B.-W.); 4 Department of Conservative Dentistry and Endodontics, Poznan University of Medical Sciences, Bukowska 70, 60-812 Poznan, Poland; maruchala@ump.edu.pl; 5 Department of Pathophysiology, Poznan University of Medical Sciences, Rokietnicka 8, 60-806 Poznan, Poland; msimon@ump.edu.pl

**Keywords:** *Polygoni cuspidati* extract, mucoadhesiveness, tablet, cyclodextrin, resveratrol, dental application, antioxidant efficacy

## Abstract

*Polygoni cuspidati* root is a resveratrol-rich source with anti-inflammatory, angiogenic and neuroprotective effects. The raw material was standardized for the content of resveratrol, for which there is a special justification for administration within the oral mucosa. To improve the solubility of resveratrol and to assure its high content in plant material, an ultrasound-assisted extraction method was applied. The addition of cyclodextrin was found to increase the extraction efficiency of resveratrol (from 13 to 297 µg per 1 g of plant material in case of 50% ethanol extracts) and enhanced its antioxidant activity as compared to pure *Polygoni cuspidati* extract/resveratrol. Cyclodextrin plays the role of a functional extract regarding technological properties (increasing the extraction of resveratrol from the extract, improving mucoadhesive properties). Therefore, the aim of this study was to develop mucoadhesive tablets containing combinations of the *Polygoni cuspidati* extract with a cyclodextrin carrier for buccal delivery. The tests sequentially included extract preparation and characterization of its physical and biological properties and then formulation studies with a broad description of the prototype properties. The test results indicate that cyclodextrin increases the efficiency of resveratrol extraction from *Polygoni cuspidati* rhizome, which is a rich source of resveratrol, and its extract enclosed in a mucoadhesive tablet guarantees prolonged action at the site of administration.

## 1. Introduction

Periodontal disease is a chronic pathological condition which, if untreated, results in damage to the dental suspension system [1]. Inflammation is mainly associated with the presence of a bacterial biofilm. However, there are also numerous other factors modifying its course (e.g., pregnancy, diabetes, endocrine, and circulatory system diseases, and the intake of certain medications). The basic idea of periodontal therapy is to control bacterial plaque, and stop the loss of the epithelial attachment and the alveolar bone [2]. In terms of the prevention and treatment of periodontal disease, non-surgical and surgical methods can be distinguished. The gold standard of the pre-surgical treatment includes scaling and root planing, which involves the removal of supragingival and subgingival dental calculus deposits [3]. In some cases, however, it is necessary to implement pharmacological treatment. Commonly used medications comprise topical antiseptics (e.g., chlorhexidine) and antibiotics or chemotherapeutic agents used systemically and locally [4]. A good therapeutic effect is also obtained by using naturally derived substances, such as resveratrol with anti-inflammatory and antioxidant properties.

One of the resveratrol-rich plant materials is *Polygoni cuspidati rhizoma et radix*. The dried root and rhizome of *Polygonum cuspidatum* (in China: Hu Zhang) belongs to Traditional Chinese medicine (TCM), and has been used to treat a variety of diseases for thousands of years. Hu Zhang contains abundant amounts of epicatechin, resveratrol, and polydatin, as well as hydrophobic components, including emodin, physcion, torachrysone and their glycosides [5]. Pharmacological data and clinical studies indicate that *P. cuspidati* extracts possess antiviral, antimicrobial, anti-inflammatory, neuroprotective, and cardioprotective activities, and the main compound responsible for pro-health action is resveratrol [5]. Emodin, in addition to its anti-inflammatory effect, has a strong constipating effect [6].

Despite a number of advantages, some phenolic compounds of *P. cuspidatum* (in particular polydatin and resveratrol) are characterized by low solubility, and high pass metabolism in the enterocytes and liver result in its low bioavailability of less than 1% [7]. Therefore, it is necessary to look for new extraction methods enabling to obtain/possess these compounds with increased solubility. Traditionally, phenolic compounds extraction from natural resources is carried out using organic solvents (e.g., methanol or ethanol). However, these extraction processes require large amounts of organic solvents associated with severe environmental problems, which limits their use. Cyclodextrin-based extraction is an emerging “green” technology of great potential. Cyclodextrins (CD) are cyclic oligosaccharides formed by six (α-CD), seven (β-CD) or eight (γ-CD) α-1,4-d-glucopyranoside units resulting in a toroid structure with interior hydrophobic cavities and exterior hydrophilic side [8,9]. CDs are increasingly used as encapsulating agents for plant biomolecules such as polyphenols to increase their water solubility, stability and bioavailability, as well as to mask their taste [10,11,12]. A recent study conducted by Gao et al. demonstrated that the use of CD plays a specific role in the extraction of components from *P. cuspidatum*, whereas the use of hydroxypropyl-β-cyclodextrin (HP-β-CD) as extractant maximized the extraction yields [13,14]. The extraction process can be enhanced by using heated reflux extraction (HRE), ultrasonic waves (ultrasound-assisted extraction, UAE) [13,15], microwaves (microwave extraction, ME), both ultrasonic waves and microwaves (ultrasound-assisted microwave extraction, UAME) [14] and supercritical phase fluids (supercritical fluid extraction (SFE)) [16].

Changing the route of administration from oral to buccal is a new approach to overcome its high-throughput enterohepatic metabolism. The oral mucosa is profoundly vascularized, absorbed active compounds straightforwardly enter the systemic circulation, bypassing the gastrointestinal tract, and first-pass hepatic digestion [17]. Such bypasses may increase the absorption of resveratrol and significantly diminish between singular inconstancy in top plasma fixation and metabolite profile to take into account more incredible clinical utility [18]. Resveratrol has high potential as a candidate for oral transmucosal absorption because it is classified to the second class of the Biopharmaceutical Classification System (BCS), with low water solubility and high membrane permeability [19]. Moreover, resveratrol has relatively small molecular weight and uncharged at physiological pH, allowing passive diffusion across the buccal mucosa [17,20]. There is some literature data about mucoadhesive pharmaceutical formulations containing resveratrol, such as lozenge [18], mucoadhesive film [21] or tablets [22].

An additional aspect of the administration of antimicrobial raw materials such as *P. cuspidatum* is the ability to maintain an appropriate oral microflora. Interaction of variable oral microorganisms helps the human body against invasion of undesirable stimulation outside. However, imbalance of microbial flora contributes to oral diseases such as dental caries, periodontitis oral mucosal diseases and is closely related to the physical state of humans, such as diabetes, obesity, and cancer. Oral microbiomes play an essential role in the human microbial community and human health [23,24]. Bacteria are the main inhabitants of the mouth and primarily comprise bacteria of the *Streptococcus mutans*, *Porphyromonas gingivalis*, *Staphylococcus*, *Lactobacillus*, and *Actinomycetes* [25,26]. Among fungi, the most important one is *Candida*, which is neutral when the oral microbiota is normal; however, when the oral microbiota balance is broken, *Candida* will seek the opportunity to attack oral tissue and forms a biofilm with *Streptococcus* (e.g., *S. mutans*) to play a pathogenic role including the development of periodontal diseases [27].

All these properties of the *P. cuspidatum* raw material contribute to its significant potential as an active agent for the supportive treatment of periodontal diseases. The spectrum of pharmacological activities of the raw material, combined with the physicochemical properties, including increased solubility resulting from the combination with CD, enables the administration of the extracts on the oral mucosa. Hence, it is fully justified to use the developed form in the treatment of inflammation, bacterial infections and vascular lesions in the oral cavity. The potential of using the developed form of dentistry is also enhanced by the possibility of masking the specific taste of the raw material by combining it with the CD. In addition, the proposed pharmaceutical form as a mucoadhesive tablet (based on mucoadhesive polymers such as (hydroxypropyl)methyl cellulose, chitosan, Carbopol and others) is to provide an increase in effectiveness, particularly in association with their prolonged contact with the mucous membranes [28,29]. Furthermore, the use of mucoadhesive formulations allows for a continuous administration of the therapeutic dose, which contributes to faster and more effective treatment of severe forms of periodontitis [30]. In addition, what is important for the patient, this form has easy and precise application, which affects the patient’s compliance [31].

To the best of our knowledge, there is no data about buccal dosage forms containing *P. cuspidati* extract.

Therefore, this work aimed to develop tablets as a novel mucoadhesive delivery platform for *Polygoni cuspidati* extract. To improve the solubility of resveratrol and to assure its high content in plant material, an ultrasound-assisted extraction method was applied and cyclodextrin was introduced into the formulation. Detailed investigations, including dissolution studies and mucoadhesive properties as well as broad in vitro assays devoted to a mode of antioxidant action of *Polygoni cuspidati* extract in combination with cyclodextrin, have been carried out.

## 2. Materials and Methods

### 2.1. Chemicals and Reagents

Plant raw material, *Polygonum cuspidatum* rhizome and root, was purchased from Herbapol Cracow (Krakow, Poland). Resveratrol (98%) (RSV) isolated from giant knotweed powder extract was supplied by PK Components (Warsaw, Poland). Emodin was obtained by Sigma-Aldrich (Poznań, Poland). Prisma™ HT buffer, Acceptor Sink Buffer, and GIT lipid solution were supplied by Pion Inc. (Billerica, MA, USA) Carbopol^®^ 974 NF polymer was obtained by Lubrizol (Wickliffe, OH, USA), whereas β-cyclodextrin, hydroxypropyl-β-cyclodextrin average Mw ~ 1460 (0.8–1.0 MS Molar Substitution) and (hydroxypropyl)methyl cellulose (HPMC) with average Mn ~ 90,000 (~15,000 cP) and magnesium stearate were supplied by Sigma-Aldrich (Poznań, Poland). The following chemicals from Sigma-Aldrich (Poznań, Poland) were used: DMSO D4540, AChE C3389, BChE C7512, ATChI A5751, BTCh B3128, DTNB D8130, donepezil D6821, neostigmine N2001, magniflorine SMB00377, rivastigmine SML0881, eserine E8375, neocuproin N1501, CuCl_2_ 307483, TPTZ 93285, Trolox 238813, fluorescein 46955, CoF2 236128, linoleic acid L1376, Tween20 P1379, β-carotene C9750, Tween 80 P1754, ascorbic acid A92902, glutathione reductase G3664, glutathione (GSH) G4251, glutathione peroxidase G6137, nicotinamide adenine dinucleotide phosphate (NADPH) N5130, ethylenediaminetetraacetic acid (EDTA) E9884, glutathione disulfide (GSSG) G4626, superoxide dismutase (SOD) S5395, nitrobluetrazolium N6639, xanthine X0626, xanthine oxidase X4875. 2,2′-Azobis(2-amidinopropane) dihydrochloride (AAPH) was from Acros Organics (401560250), cyclooxygenase-2 (COX-2, human recombinant, 60122) and COX-2 activity assay kit (760151) were from Cayman Chemicals (Ann Arbor, MI, USA). Buffer salts, solvents and other reagents were from Sigma Aldrich (Poznań, Poland) and were at least of analytical grade. HPLC grade acetonitrile was obtained by Merck (Warsaw, Poland). High-quality pure water and ultra-high-quality pure water were prepared by using a Direct-Q 3 UV Merck Millipore purification system. Buccal mucosa from the porcine cheek was obtained from Bost slaughterhouse (Turosn Koscielna, Poland). Samples excised immediately after animal death were washed and frozen at −20 °C in isotonic saline solution. Prior to experiments, tissue was defrosted at ambient temperature and cut into pieces.

### 2.2. Preparation and Analysis of Polygoni cuspidati Extract—Preformulation Studies

#### 2.2.1. Extract Preparation and Freeze-Drying

A total of 5.0 g of ground dry plant raw material was placed in a conical flask and extracted with 50.0 mL of a mixture of ethanol and distilled water (3:7, 5:5, 7:3 or 10:0 *v*/*v*) at the temperature of 65 °C in an ultrasonic bath, four times (each time using fresh extraction mixture) for 20 min. Extracts were concentrated to 50.0 mL using an evaporator, and marked as W1–W14 (Table 1). The addition of β-CD and HP-β-CD to the extraction mixture (in the proportion indicated in Table 1; by mixing cd with extraction mixture at the room temperature for 30 min) was used as a extraction process modifier and RSV and emodin encapsulating factor.

Then the extracts were frozen and lyophilized (Heto PowerDry PL3000 Freeze Dryer, Thermo Scientific, Waltham, MA, USA). The condensation temperature was set to −55 °C. The freeze-drying was conducted at reduced pressure (6.2 hPa) for 48 h.

#### 2.2.2. Determination of Resveratrol and Emodin Content in Extracts

The concentrations of main active compounds (RSV and emodin) were determined by using the modified HPLC-Diode-Array Detection method described previously by Qian et al. [32] using LC system (Dionex Thermoline Fisher Scientific, Waltham, MA, USA) with Chromeleon software 7.0 version. Separations were carried out on a LiChrospher RP-18 column with 5 μm particle size, 250 × 4 mm (Merck, Darmstadt, Germany). A diode array detector was used to detect the wavelength maxima (λ_max_) of 290 nm.The following modifications in Qian’s method were done: The mobile phase was composed of formic acid 0.1% (A) and acetonitrile (B) with a gradient elution of 0–20 min, 15–20% B; 20–40 min, 20–40% B; 40–60 min, 40–100% B; 60–65 min, 100% B; 65–70 min, 15% B. The flow rate of the mobile phase was 1.0 mL/min and the column temperature was maintained at 30 °C.

The presence of RSV and emodin in the extract was confirmed by comparing retention time and UV spectra of substances presented in the extract with their reference standards. The International Conference on Harmonization Guideline Q2 was used to validate the HPLC-DAD method and included selectivity, linearity, intra- and inter-day precision, limits of detection (LOD) and quantitation (LOQ) [33].

#### 2.2.3. Dissolution Studies

An Agilent 708-DS dissolution apparatus was used to study the dissolution rate of resveratrol and emodin from cyclodextrin-based lyophilized extracts. A standard paddle method was used at 37 ± 0.5 °C with a stirring speed of 50 rpm. Around 200 mg of extracts were weighed into gelatin capsules and placed in a sinker to keep the capsule from floating on the liquid’s surface. The systems were submerged in 900 mL of pH 6.8 phosphate buffer. At predetermined intervals, liquid samples were obtained and replaced with an equivalent volume of temperature-equilibrated medium. A 0.45 m nylon membrane filter was used to filter the samples. The above-described HPLC procedure was used to determine the RSV and emodin concentration in the filtered acceptor solutions. In the studies, sink conditions were maintained.

Moore and Flanner’s model, which is based on two-factor values, *f*_1_ and *f*_2_, was used to compare release profiles. According to the formulas below, the difference factor (*f*_1_) is a logarithmic transformation of the sum-squared error of differences between the test *T_j_* and reference *R_j_* system over all time points, and *f*_2_ is a logarithmic transformation of the sum-squared error of differences between those systems [34]:

$$f_{1}=\frac{\sum_{j=1}^{n}\left|R_{j}-T_{j}\right|}{\sum_{j=1}^{n}R_{j}}\times 100\;f_{2}=50\times \log\left({\left(1+(\frac{1}{n})\sum_{j=1}^{n}{\left|R_{j}-T_{j}\right|}^{2}\right)}^{-\frac{1}{2}}\times 100\right)$$

where *n* is the sampling number, *R_j_* and *T_j_* are the percentages dissolved of the reference (RSV) and test products (RSV/CD systems) at each time point *j*. Dissolution profiles are similar when the *f*_1_ value is close to 0 and *f*_2_ is close to 100.

#### 2.2.4. Permeability Studies

Using a PAMPA GIT model to simulate the gastrointestinal tract, the permeability through an artificial biological membrane of RSV and emodin was examined. A 120-µm-thick microfilter disc coated with a 20% (*w*/*v*) dodecane solution of a lecithin mixture separated two chambers: a donor at the bottom and an acceptor at the top (Pion, Inc., Billerica, MA, USA). The standards were dissolved in the donor solution at pH 6.8. The plates were assembled and incubated in a humidity-saturated environment at 37 °C for 3 h. The HPLC-DAD method was used to determine the RSV and emodin concentration in the donor and acceptor compartments. The apparent permeability coefficients (P_app_) were determined using the equation below:

$$P_{\mathrm{app}}=\frac{-\ln\left(1-\frac{C_{A}}{C_{\mathrm{equilibrium}}}\right)}{S\times \left(\frac{1}{V_{D}}+\frac{1}{V_{A}}\right)\times t}$$

where V_D_—donor volume, V_A_—acceptor volume, C_equilibrium_—equilibrium concentration $C_{\mathrm{equilibrium}}=\frac{C_{D}\times V_{D}+{\;C}_{A}\times V_{A}}{V_{D}+V_{A}}$, C_D_—donor concentration, C_A_—acceptor concentration, S—membrane area, t—incubation time (in seconds).

An ANOVA test was utilized to ensure that P_app_’s permeability determination was statistically different. Compounds with P_app_ < 1 × 10^−6^ cm/s are classified as low-permeable and those with P_app_ > 1 × 10^−6^ cm/s as high-permeable compounds [35].

#### 2.2.5. Total Polyphenolic Compounds—Folin-Ciocalteu Method

25 μL of the extract (in the concentration range 0.31–5.00 mg/mL) or gallic acid solution (in the concentration range 6.25–100.00 µg/mL) to be tested were transferred to the wells of a 96-well plate. Then 200 μL of distilled water, 15 μL of Folin-Ciocalteu reagent and 60 μL of 20% sodium carbonate solution were added. The blank was a mixture of 25 μL of distilled water (for gallic acid) or DMSO (for extracts) and other reagents. The plate was wrapped in aluminum foil, shaken for 5 min (25 °C, 600 rpm) and incubated for 25 min at room temperature. The absorbance was measured using a UV plate reader at λ = 760 nm. The total gallic acid content in the tested preparations was calculated on the basis of the calibration curve prepared for the standard substance and expressed as gallic acid equivalent [mg GAE/g plant material].

#### 2.2.6. Antioxidant Activity

##### Assay with 2,2-Diphenyl-1-Picrylhydrazyl (DPPH)

According to Kikowska et al. [36], the DPPH assay was performed. In a nutshell, 25 μL of extracts dissolved in DMSO at various concentrations (0.19–6.25 mg plant material per mL for liquid extracts or 0.03–0.50 mg for lyophilized extracts) were combined with a 175 μL of *DPPH* solution (3.9 mg/50 mL of MeOH). The reaction solution was stirred and incubated for 30 min in the dark at room temperature. Absorbance was measured at 517 nm against the blank (25 μL of DMSO and 175 μL of MeOH). 25 μL of DMSO and 175 μL of *DPPH* solution made up the control sample. The percent of *DPPH* scavenging activity was determined using to the formula below:

$$DPPH_{\mathrm{scavenging}\mathrm{activity}\;}\left(\%\right)\;=\frac{A_{0}-A_{1}}{A_{0}}\times 100\%$$

where *A*_0_ is the absorbance of the control, and *A*_1_ is the absorbance of the sample.

Six replicates of the analyses were carried out. The results were represented as the IC_50_ value, which was calculated using the quadratic equation and corresponds to the RSV concentration required to inhibit DPPH radical production by 50%.

##### Cupric Reducing Antioxidant Capacity (CUPRAC) Assays

The CUPRAC assay was performed with modifications according to Apak et al. [37]. Equal quantities of 7.5 mM neocuproine solution in 96% ethanol, acetate buffer (pH = 7.0), and 10 mM CuCl_2_·H_2_O solution were used in the CUPRAC reagent solutions. In a short, 50 μL of extracts dissolved in DMSO at various concentrations (0.194–6.25 mg plant material per mL for liquid extracts or 0.03–0.50 mg for lyophilized extracts) were mixed with 150 μL of CUPRAC solution, mixed, and incubated for 30 min in the dark condition at room temperature. After that, the absorbance was measured at 450 nm. Six replicates were used in the analysis. The results were represented in terms of the IC_0.5_, which is the extract concentration required to achieve an absorbance value 0.5.

##### Ferric Antioxidant Power (FRAP) Assays

After mixing the sample (0.02 mL) with 1.9 mL of FRAP solution for 30 min at room temperature, the absorbance was measured at 593 nm, after shaking for 30 s. The FRAP solution was made by mixing 2.5 mL 5 mM TPTZ solution (prepared in 40 mM HCl solution), 2.5 mL 5 mM FeCl_3_ solution and 25 mL acetate buffer (0.3 M, pH 3.6), and warming in a water bath at 37 °C for 20 min. Instead of the sample, a blank (reagent) sample was created with buffer, and the sample’s background was examined (a mixture containing studied sample and buffer only). A total of 0.51 mg of Trolox dissolved in 1 mL DDI water (stock solution diluted to obtain 20 standard solutions in the range of 0.0255–0.51 mg Trolox/mL) was used to create the calibration curve [38].

##### Oxygen Radical Absorbance Capacity (ORAC) Assay

The sample (10 μL) was mixed with 170 μL of fluorescein (0.00020941 mg fluorescein/10 mL 75 mM phosphate buffer, pH 7.4) and incubated for 20 min at 37 °C. The fluorescence was read (excitation at 485 nm and emission at 520 nm) at the start and every 1 min during the entire reaction with steady shaking, till stabilization. Instead of the sample, a blank sample containing phosphate buffer was run. In addition, the background from the samples was measured (a mixture containing studied sample and DDI water only). Activity was expressed in Trolox equivalents prepared using 12 dilutions of a stock solution (50 μM Trolox/L) [39].

##### Effect on Superoxide Dismutase (SOD) Activity

The sample (0.05 mL) was combined with 10 μL SOD (0.24 U), 160 μL nitrobluetatrazolium solution (0.0025 M), 205 μL phosphate buffer (0.2 M, pH 7.5), 30 μL xanthine (150 mM in 1 M NaOH) and 0.01 mL xanthine oxidase (0.065 U). After 20 min of incubation, the change in absorbance at 550 nm in tested samples vs. controls without investigated sample was measured, and the effect on the enzyme was calculated using equation [40]:

$$Inhibition\left(\%\right)\;=100-100\times \frac{A_{30min}-A_{0min}}{A_{control\;30min}-A_{control\;0min}}$$

##### Hydroxyl Radical Averting Capacity (HORAC) Assay

Fluorescein solution (170 L, 60 nM) was combined with the sample (0.01 mL) and incubated at 37 °C for 10 min. Then, to the tested sample, 10 L of 27.5 mM H_2_O_2_ solution and 10 L of CoF_24_H_2_O solution (230 M, containing 1 mg of picolinic acid/mL) were added. The fluorescence was measured at the beginning and every 1 min until the reaction stabilized (excitation at 485 nm and emission at 520 nm) (typically 5–10 min). Instead of the sample, the blank sample contained phosphate buffer. In addition, the background from the samples was measured (a mixture containing studied sample and DDI water only). Gallic acid equivalents (GAE) were calculated using 15 gallic acid solutions (9.6–480.0 g of gallic acid/mL), as stated previously [39].

##### Effect on Glutathione Reductase (GR) and Glutathione Peroxidase (GPx) Activity

The following procedure was used to test the effect on glutathione reductase (GR). The sample (0.02 mL) was mixed with 10 mL EDTA solution, 12 mL GSSG solution, and incubated for 5 min at 25 °C before adding 4 mL NADPH solution (all reagents were diluted in 0.1 mM sodium phosphate buffer, pH 7.6) and recording the first absorbance (340 nm). The reaction was then begun by adding 2 U glutathione reductase (2 L, Sigma Aldrich number G3664), 177 L of 0.1 mM sodium phosphate buffer, and recording the absorbance after 5 min at 25 °C. Concentrations of reagents in the final solution (805 μL) were as follows: 0.5 mM EDTA, 10 mM GSSG and 10 mM NADPH. Instead of the sample, a blank sample was made with buffer. In addition, the background was measured (mixture containing studied sample and buffer only). In comparison to nmol of NADPH consumed/min in blank (reagent) sample, one unit of enzyme activity has been defined as nmol of NADPH consumed/min × mL sample [41].

The following procedure was used to test the effect on glutathione peroxidase (GPx). Sample (0.020 mL) was mixed with 8 μL of EDTA solution, 10 μL of glutathione reductase (0.2 U Sigma G3664), 4 μL of GSH solution, 10 μL of glutathione peroxidase (0.04 U Sigma G6137), 22 μL of H_2_O_2_ and 332 μL of 50 mM sodium phosphate, pH 7.0). To begin the reaction, 4 μL of NADPH solution (N5130) was added, and after 10 min of incubation at 25 °C, the decrease in absorbance (340 nm) was measured. All solutions were made in a 50 mM buffer, with the following reagent concentrations in the final mixture—1.5 mM H_2_O_2_, 0.04 U glutathione peroxidase, 1 mM EDTA, 0.2 U glutathione reductase, 2 mM GSH, 0.04 U glutathione peroxidase, 0.8 mM NADPH Instead of the sample, a blank sample was created using buffer, and the background was assessed (mixture containing studied sample and buffer only). In comparison to nmol of NADPH consumed/min in the blank (reagent) sample, one unit of enzyme activity was defined as nmol of NADPH consumed/min × mL sample [42].

##### Inhibition of Lipid Peroxidation

The conjugated diene technique was used to measure antioxidant activity in a linoleic acid model system (linoleic acid oxidation test). Linoleic acid (800 mg) was freshly dissolved in 20 mL pure MeOH, then combined with 200 mL 0.2 M sodium phosphate buffer (pH 6.5) and Tween 20. (6.5 mM conc. of Tween 20 was obtained). For 10 min, the emulsion was sonicated. 0.2 mL of the sample was combined with 1.8 mL of linoleic acid emulsion and incubated at 37 °C. After 4 h of incubation, samples (0.1 mL) were obtained and mixed with 1.2 mL of 100% MeOH. At 234 nm, the absorbance was measured against a blank sample without the examined solution. In the same way as the tested samples, ten ascorbic acid solutions (223.5–1676.3 g ascorbic acid/mL) were used to create the calibration curve. The sample’s background was measured at 234 nm (a mixture containing studied sample and buffer) [43].

##### Beta-Carotene Bleaching Test

β-Carotene (7 mg) was combined with 350 mL linoleic acid and 2.8 g Tween 80 in 5 mL chloroform. Under vacuum (40 °C), chloroform was evaporated, and 100 mL of DDI water containing oxygen was added, followed by vigorous shaking. The β-carotene/linoleic acid emulsion (200 µL) was combined with the sample (200 µL). At 463 nm after 4 h at 50 °C, the absorbance at zero time and the change in absorbance were determined. Instead of the studied samples, a stock solution of ascorbic acid (0.94 mg/mL) was created, followed by the fabrication of series dilutions (9.4–94 g/L). The background of the samples was determined (a mixture containing studied sample and DDI water only). The percent activity of samples was determined using blank samples containing only emulsion and DDI water [44].

#### 2.2.7. Effect on Cholinesterase (ChE) Activity

The colorimetric approach of Ellman [45] was employed, with some adjustments [46]. Tested sample (10 μL) was combined with 20 μL of AChE (or BChE) solution (0.28 U/mL) and after 5 min 35 μL of ATChI (or BTCh) (1.5 mM/L), 175 μL of 0.3 mM/L DTNB (containing 10 mmol/L NaCl and 2 mM/L MgCl_2_) and 110 μL with Tris-HCl buffer (50 mM/L, pH 8.0) was added. In place of the examined sample, samples containing 35 μL of Tris-HCl buffer were run in the same way (“blank” samples). Using “blank” samples containing ATCh (or BTCh) and DTNB completed to 345 µL with Tris-HCl buffer, the increase in absorbance owing to spontaneous hydrolysis of the substrate was observed. The absorbance was measured (405 nm, 96-well microplate reader, Tecan Sunrise, Männedorf, Switzerland) after all samples were incubated at 22 °C for 30 min (incubation time was calculated after optimization studies, details not shown). According to Rhee et al., the “false-positive” effect of the chemicals examined was measured [47] with minor modifications, as described previously [46]: After mixing of the substrate with the enzyme and buffer, the “false-positive” sample was left for incubation. Then an examined sample and DTNB were added, and the absorbance was measured right away.

The results were calculated using reference cholinesterase inhibitors (eserine, neostigmine, magniflorine, rivastigmine and donepezil). For this, 16 dilutions in pure DMSO (2.57–41.14 µg/mL) were made for each chemical. These solutions (10 µL) were tested and calibration curves were created as mentioned above.

All solutions utilized in a series of assays were produced in the same buffer, and each sample was evaluated at least eight times. The background of the sample (10 L mixed with 365 L of Tris buffer) was measured at 405 nm for calculations and removed. The test sample’s absorbance was then subtracted from the “blank” sample’s absorbance.

#### 2.2.8. Anti-Inflammatory Activity

##### Effect on Cyclooxygenase-2 (COX-2) Activity

For the assay, chemicals from the Cayman COX-2 assay kit were prepared according to the manufacturer’s instructions and mixed with COX-2 enzyme (human recombinant, Cayman No. 60122, pre-diluted 100-fold using 100 mM, pH 8.0 Tris buffer). A volume of 0.01 mL of the examined sample was combined with 0.12 mL of Tris buffer (100 mM, pH 8.0), 0.01 mL hemin, and shaken for 5 min at 25 °C before adding 0.02 mL colorimetric substrate and 0.02 mL arachidonic acid solution. 0.02 mL COX-2 solution was added to start the reaction. The increase in absorbance during the room temperature incubation was measured at 590 nm. Positive (assay kit COX-2 inhibitor DuP-697) and negative (blank) samples (buffer instead of examined sample) were run at the same time. The background of the studied samples (0.04 mL sample + 0.19 mL buffer) was also measured and accounted for in the calculations. Each sample was tested at least four times. The percentage of inhibition of enzyme activity was calculated (indicates by how many percent the activity has been reduced in relation to the negative-blank sample for which the maximum activity was assumed as 100%, under the conditions used in the method). The acetylsalicylic acid equivalent concentration (mg/mL) was also used to express the inhibition of enzyme activity. Acetylsalicylic acid solutions were produced at 14 concentrations (0.2–10 mg/mL) for this purpose and examined in the same way as the tested samples.

### 2.3. Formulation Studies

Powder binary systems in weight ratio 1:1 (*w*/*w*) of *P. cuspidati* lyophilized extract L5 (with the highest biological activity based on the above-mentioned research) and the following excipients: Carbopol^®^, HPMC and magnesium stearate were prepared. Binary systems with a weight ratio from predefined formulation were also created (Table 2). These systems were tested for stability at 25 ± 2 °C and controlled air humidity (RH = 50%). The concentrations of active substances were determined using the HPLC-DAD method at the appropriate time intervals (3, 6, and 12 months).

Additionally, the antioxidant properties of the formulation composition in powder systems (Table 2) were investigated (according to the methodology in Section 2.2.5 and Section 2.2.6).

#### 2.3.1. Identification of Tableting Powder Blends—Fourier Transform Infrared Spectroscopy with Attenuated Total Reflectance (FTIR-ATR)

The FTIR-ATR spectra were measured between 400 and 4000 cm^−1^, with a resolution set to 1 cm^−1^, with a Shimadzu IRTracer-100 spectrometer equipped with a QATR-10 single bounce—diamond extended range and LabSolution IR software.

#### 2.3.2. Tableting Process

A laboratory scale, single punch tableting machine, the NP-RD10A Tablet Press, was used to compress flat-faced, 8 mm in diameter tablets (Natoli). Tablet compaction properties were evaluated using a variety of compaction pressures ranging from 60 to 100 MPa. When the desired compaction pressure was reached, the pressure was released. Table 2 lists the ingredients in the designed tablets.

##### Tablet Characterization

The dimensions and weight of newly created tablets were recorded as soon as they were compacted. The tablets’ weight homogeneity was determined using the method provided in Ph.Eur. 10th [48]. A manual vernier caliper was used to measure the thickness and diameter of 20 tablets at random. The mean values and standard deviations of all measurements were calculated (SD).

The hardness of the tablets was determined using the PTB-M manual tablet hardness testing instrument (Natoli), according to the methods provided in Ph.Eur. 10th. Each hardness rating is derived from the average of six measurements and expressed as a mean with standard deviation (SD).

The breaking force (F) values [N] were used to compute the tensile strength (*σ*), where *d* is the diameter of the tablet [mm] and *h* is the thickness of the tablets [mm] [49].

$$\sigma =\frac{2F}{\pi dh}$$

The tablet porosity (*ε*) was calculated using the following equation:

$$\varepsilon =1-\frac{W_{t}}{\rho_{true}v}$$

where *W_t_* was tablet weight [mg], *v* was volume of tablet and *ρ_true_* was true density of the powder [g/cm^3^].

#### 2.3.3. Swelling Index

Weighted tablets were placed separately in a 50 mL beaker containing 25 mL of pH 6.8 phosphate buffer. The beakers were kept at 37.5% of their original temperature. Samples were withdrawn, wiped off with filter paper, and reweighted at predefined time intervals (1, 2, 3, 4, 5, and 6 h).

The following formula was used to determine the swelling index:

$$SI=\frac{W_{2}-W_{1}}{W_{1}}$$

where *SI* is the swelling index, *W*_1_ is the initial weight of the tablet, *W*_2_ is the weight of the tablet after the particular swelling time interval.

Each experiment was performed in triplicate.

#### 2.3.4. In Vitro Release Studies

Studies were conducted using a standard paddle method at 37 ± 0.5 °C with a stirring speed of 50 rpm. The tablets were placed in 900 mL of phosphate buffer at pH 6.8. Sink conditions were maintained thorough the studies.

The collected active compounds release profiles were fitted to the following mathematical models in order to explore the release kinetics:

zero order equation: F=k×t,
first-order equation: lnF=k×t,
Higuchi equation: $F=kt^{1/2}$,
Korsmeyer-Peppas equation: $F=kt^{n}$,

where *F*—the fraction of release drug, *k*—the constant connected with release and *t*—the time.

#### 2.3.5. Mucoadhesive Properties

##### In Vitro Assessment of Mucin-Biopolymer Bioadhesive Bond Strength

The bioadhesive binding strength between mucin and chitosan was measured using a viscometric technique. The assessment was carried out according to Hassan and Gallo’s procedure [50].

The viscosity coefficient of a hydrophilic dispersion including mucin and bioadhesive polymers HPMC and Carbopol^®^ was computed using the equation below:

$$\eta_{t}=\eta_{m}+\eta_{p}+\eta_{b}$$

where *η_t_* is the viscosity coefficient of the system, and *η_m_* and *η_p_* are the individual viscosity coefficients of mucin and bioadhesive polymer, respectively, and *η_b_* is the viscosity of component due to bioadhesion and can be obtained by rear-ranging above equation:

$$\eta_{b}=\eta_{t}-\eta_{m}-\eta_{p}$$

The force of bioadhesion, *F*, represents the additional intermolecular frictional force per unit area and was determined by:

$$F=\eta_{b}\times \sigma$$

where *σ* is the rate of shear per second.

##### Determination of the Mucoadhesive Behavior in Contact with Porcine Buccal Mucosa (Maximum Detachment Force and Work of Adhesion)

The mucoadhesive potential of formulations F1–F6 in contact with porcine buccal mucosa was determined using tensometric method on texture analyzer TA.XT Plus (Stable Microsystems, Godalming, UK). Performed experiments did not require the approval of Local Ethic Committee. The porcine tissue was glued to thermostated stainless steel plate and warmed at 37.0 ± 2 °C for 5 min. Each tablet was moisturized with 50 µL of phosphate buffer (pH 6.8) and adhered with a glue to the upper A/muc probe. Subsequently, the probe/tablet was lowered with a speed of 2 mm/s to make contact with a tissue specimen for 60 s (contact force was established at 0.3 N) and then returned with a constant speed of 2 mm/s. The maximum detachment force necessary to separate the tablet from the tissue sample was recorded directly whereas the work of mucoadhesion was calculated from the area under the force versus distance curve. The studies were carried out at least in quintuplicate.

##### Determination of the Residence Time

The residence time of formulations F1–F6 to porcine buccal mucosa was evaluated on adjusted apparatus for the disintegration time test according to Nakamura et al. [51]. The segments (2–3 cm length, 0.2 cm thickness) of tissue from porcine cheek were glued with mucosal surface faced upwards to the inner surface of the glass beaker. The medium simulated saliva was phosphate buffer (pH 6.8, 500 mL) maintained at 37 ± 2 °C. Each tablet was bought into a contact with tissue specimen by putting on a finger force for 5 s. The experiment began when a plexiglass tube (diameter 6 cm, weight 280 g) fixed to the apparatus started moving up and down continuously. During the test each attached formulation was entirely immersed in a buffer at the lowest point of tube location and was out at the highest point of tube location. The time necessary to detach formulation from the mucosal tissue was measured within 3 h of performed test. Studies were carried out in triplicate.

## 3. Results and Discussion

As the first stage of the experimental work, hydroalcoholic liquid and freeze-dried extracts were prepared (solid content of lyophilized extracts presented in Supplementary Materials, Table S1). The use of ultrasound-assisted extraction with cyclodextrins as extraction modulators allowed to obtain extracts with different RSV and emodin content. In connection with the targeted administration of the prepared extracts through the oral mucosa, it was necessary to obtain an extract with a high RSV and low emodin content to eliminate its constipating effect.

**Figure 1:**
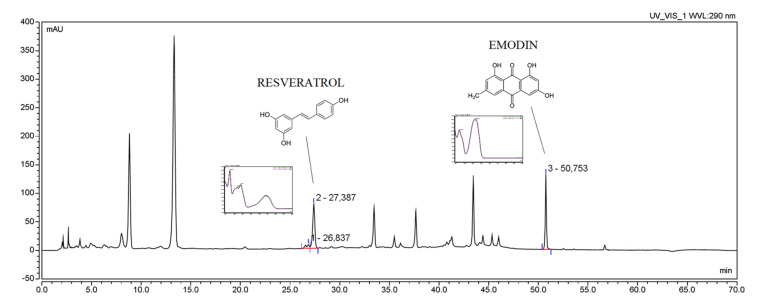
The HPLC chromatogram of *Polygoni cuspidati* rhizome and root extract L5 (concentration 5 mg/mL) with selected patterns of resveratrol and emodin.

The active compounds (RSV and emodin) present in the plant material and *P. cuspidati* rhizome and root liquid and lyophilized extracts, as well as all concentration changes of RSV and emodin during dissolution and permeability studies, were identified and determined using a high-performance liquid chromatography system with a photodiode array detector. The HPLC-DAD method was developed according to the method described Qian et al. [32], which was modified regarding the gradient elution, and then validated according to ICH Q2 guidelines. Supplementary Materials contain validation parameters (Table S2). Initially, the method was developed on three standards (RSV, emodin, parietin), but due to the low concentration of parietin in the prepared extracts, it was decided to validate the method only for two standards (RSV and emodin) (Figure 1). No significant change was observed in the retention time of individual peaks depending on the extract preparation method. The developed method may be the reference method for determining the concentration of resveratrol and emodin in plant material and pharmaceutical dosage forms containing those active compounds.

Using the linearity equation of reference substances, it was possible to determine the content of active compounds in the lyophilized extract. The content of compounds in all tested liquid and lyophilized extracts is presented in the Supplementary Materials, Table S3. As the concentration of ethanol in the extraction mixture increased, the amount of extracted resveratrol increased, for example 44 µg/1 g plant material for W1 based on 30% ethanol vs. 401 µg/1 g of plant material for W12 based on 100% ethanol. More importantly, with the increase in the concentration of ethanol, the amount of isolated emodin increased several times (0.56 µg/1 g of plant material for W1 based on 30% ethanol vs. 354 µg/1 g of plant material for W12 based on 100% ethanol), which is a potentially constipating ingredient, so its significant amount in the prepared extract does not was desirable from the point of view of buccal administration. Therefore, the more critical parameter was the ratio of resveratrol and emodin content. The best ratio of resveratrol and emodin content was found in the W5 and L5 extracts and contained 5.95 μg of resveratrol per 1 g of plant material and 0.27 μg of emodin per 1 g of plant material for W5, and 1.87 μg of resveratrol per 1 mg of lyophilized extract and 1.91 μg of emodin per 1 mg of lyophilized extract for L5, and, therefore, L5 was selected for the part of the formulation studies.

**Figure 2:**
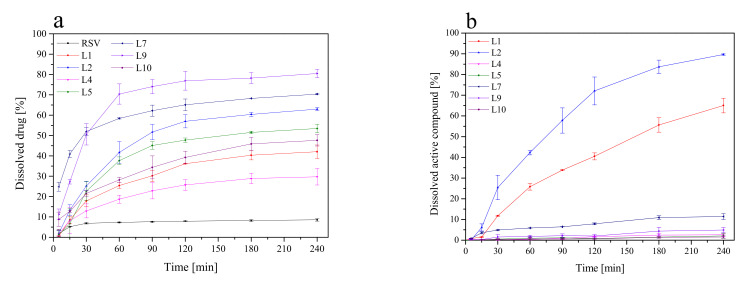
Dissolution profiles of the RSV (**a**) and the emodin (**b**) from lyophilized extracts at pH ~ 6.8.

In vitro dissolution studies of selected lyophilized extracts were performed at pH = 6.8, simulating the conditions of the oral cavity environment to compare the changes in dissolution rates of RSV and emodin. Figure 2 shows the dissolution rate profiles for both active compounds. Due to the lack of availability of the emodin standard, it was not possible to perform a dissolution rate test for it. The dissolution of emodin from the lyophilized extracts was only compared.

The highest dissolution rate of active compounds was observed for lyophilized extracts where CDs were added as solubilization enhancers (L5). As we can see in Figure 2b, the dissolution rate of emodin from L1 and L2 was high and reached approximately 70% and 90% (whereas relatively low RSV release was observed for these formulations), which point toward potential constipation effect after taking this compound; thus formulation L1–L2 were excluded from further studies. In turn, formulation L5 displays a relatively high concentration of RSV in acceptor media and simultaneously low concentration of dissolved emodin and therefore was selected for further analysis.

The buccal route of drug administration serves a potential increase in the bioavailability of resveratrol. It was essential to check the permeability of both resveratrol and emodin from prepared liquid and lyophilized extracts. All apparent permeability values are collected in the Supplementary Materials, Table S4. Interestingly, the permeation of RSV and emodin was higher in the studies using extracts than with the standard itself. It is related to the ‘entourage effect’, which has been previously described for other plant materials [52]. RSV permeation from L5 was slightly lower as compared to formulations L1, L3, L9–L11, while in the case of L5 a favorable low emodin permeation was observed, which confirms the selectivity of cyclodextrin-based extraction.

The antioxidant properties of *P. cuspidati* extract have been widely described in literature data [53,54,55]. Comparing previous antioxidant data, extract W5 exhibited higher antioxidant properties than literature data (IC_50_ = 0.22 for W4 vs. 24.95 μg/mL in case of water-ethanol extracts 50/50 *v*/*v*) [55]. The water-ethanol extracts W5 prepared in this work, despite the total lower polyphenolic compounds content (54.5 for W5 vs. 641.1 mg/1.0 g material for prepared by 50% ethanol solution at 25 °C for 30 min with shaking), showed higher activity in DPPH assay than the ethanol extract (IC_50_ = 0.16 vs. 110 μg/mL in case of liquid extract) [53]. All data from in vitro antioxidant studies confirmed both free radical scavenging as well as metal-chelating activity (Supplementary Materials, Tables S5–S7). In addition to the classic antioxidant activity, the presented study showed the ability to inhibit butyrylcholinesterases (BChE), which in turn may indicate the possibility of using the extracts in the prevention of neurodegenerative diseases (Table S8). First of all, cyclodextrins have been found to have poor own antioxidant properties, nevertheless can increase the antioxidant potential of systems with other compounds [56]. Higher activity of extracts prepared with cyclodextrin can be explained because some OH groups of the polyphenols could be further protected in the β-CD cavity [56], which is in line with finding based on (e.g., resveratrol–cyclodextrin interactions) [57]. Moreover, cyclodextrins have been found to prevent food browning and improve the antioxidant capacity of foods [58]. Interestingly, the whole set of active compounds is responsible for the antioxidant effect of the extract, not just one selected element, which is visible in the results (e.g., for resveratrol itself). Assuming that only the aforementioned resveratrol would be responsible for the antioxidant activity of the extract, converting its activity into its content in the extract, the extract activity should be several dozen times lower. This is the proof that there is a group of compounds that contribute to the total activity of the prepared extracts. It is called ‘entourage effect’, and it has been extensively described for cannabinoids [59].

In addition to the antioxidant activity of the extracts as well as RSV and emodin, there are reports on its anti-inflammatory activity, expressed by inhibiting cyclooxygenase. Presented data displayed the antioxidant activity of extract and the highest values were obtained for lyophilized extracts L4, L6 and L10 (Supplementary Materials, Table S9). The literature data emphasizes that resveratrol, despite unfavorable pharmacokinetics, can modulate the inflammatory pathways underlying neurodegenerative, respiratory, metabolic and cardiovascular diseases [60] due to non-selective inhibition of COX-2 [61]. Moreover, also isolated emodin has anti-inflammatory effects [62,63], which further explains the activity of the prepared extracts.

Obtained extract with a high RSV and simultaneously low emodin content (L5) characterized by increased dissolution rate and permeability of RSV and profound antioxidant activity was chosen for further formulation studies aimed at developing dosage form for buccal delivery of *Polygoni cuspidati* extract. The precise goal was to assure mucoadherence to the mucosal tissue.

Six tablet formulations F1–F6 with Carbopol^®^ 974 NF and HPMC as mucoadhesive agents were prepared (Table 2). All formulations had a constant percentage of lyophilized cyclodextrin-based *Polygoni cuspidati* extract (80.0 mg), and magnesium stearate (1% of final tablet weight) as lubricant.

**Figure 3:**
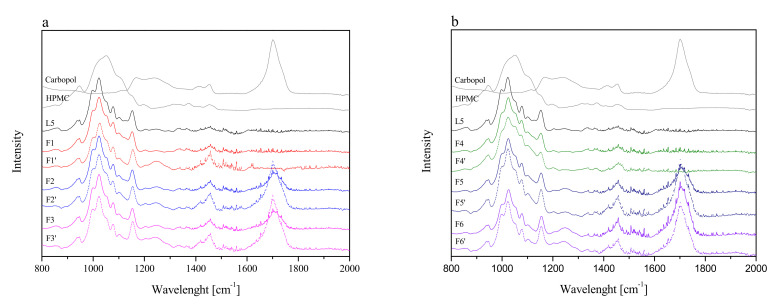
FTIR-ATR spectra of lyophilized extract L5 and formulations F1–F3 (**a**) and F4–F6 (**b**), where F1–F6 corresponds to formulations F1–F6 and F1′–F6′ corresponds to the theoretical sum of the spectra of the lyophilized extract L5 and the excipient base used to prepare the formulation F1–F6.

Firstly, tableting powder blends were identified by using FTIR-ATR spectroscopy. To determine whether new interactions between the extract and excipients during the tableting of the powder blends occurred, spectra of lyophilized extract L5 and formulations F1–F6 were taken and presented in Figure 3. Additionally, Figure 3 shows the theoretical sum of the spectra of the lyophilized extract L5 and the excipient base used to prepare the formulations F1–F6. Comparing the F1–F6 and F1′–F6′ spectra, no additional bands or shifting of existing bands were observed, which may indicate no intermolecular interactions between the extract and the excipients [64].

**Figure 4:**
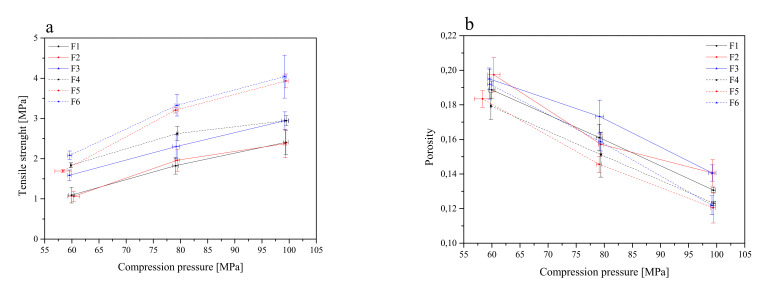

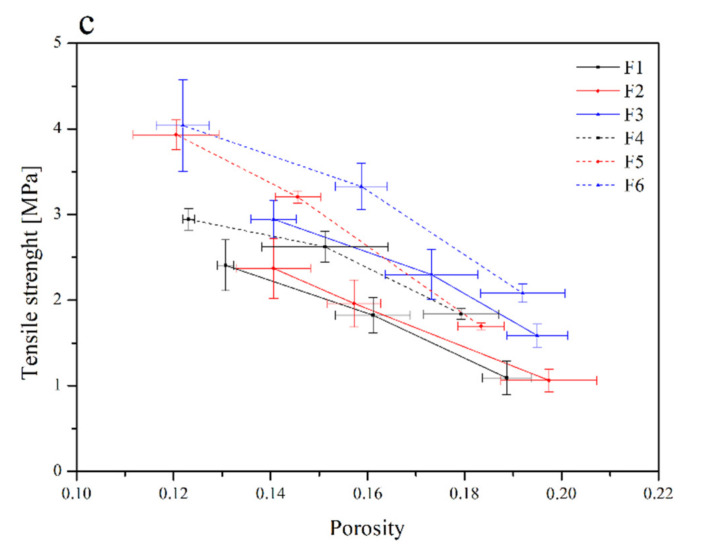
Tabletability (**a**), compressibility (**b**) and compactability (**c**) profiles of the tablets containing lyophilized cyclodextrin-based *Polygoni cuspidati* extract (formulations F1–F6).

Then, flat-face tablet formulations F1–F6 were prepared (Table 2) and initially characterized in terms of tabletability, compressibility and compactability (Figure 4).

The tabletability of the tablets decreased in the following order: F6 > F5 > F4 > F3 > F2 > F1. Such an order is related to the composition of the individual components. In general, it can be said that tablets with the composition Carbopol^®^ and HPMC in the ratio of 1:1 (F3 and F6) showed better tabletability properties than the composition Carbopol^®^ and HPMC in the ratio of 1:2 (F2 and F5). The least desired properties were observed for HPLC-based tablets (F1 and F4) (Figure 4a). However, Carbopol^®^/HPMC-based tablets showed significantly higher crushing strength than HPMC-based tablets. This indicates that the Carbopol^®^ polymer has better binding characteristics. The general trend of the compressibility profile showed that as the pressure load applied to the powder samples decreased, the porosity level or the solid fraction value increased (Figure 4b). The tablets F3 and F2 showed better compressibility as relatively high porosity was retained at higher compression values. A powder’s compactability is defined as its ability to form coherent, strong compacts [65], and it is obvious that less dense tablets have greater porosity, due to the higher number of pores in the tablet resulting in poor interparticle bonding, and thus, the tablet requires a lower force to break down. The order of decreasing compactability appears to be as follows: F6 > F5 > F3 > F4 > F2 > F1 (Figure 4c). Based on the above parameters, the best tablet properties were obtained for formulations F6 and F5.

**Figure 5:**
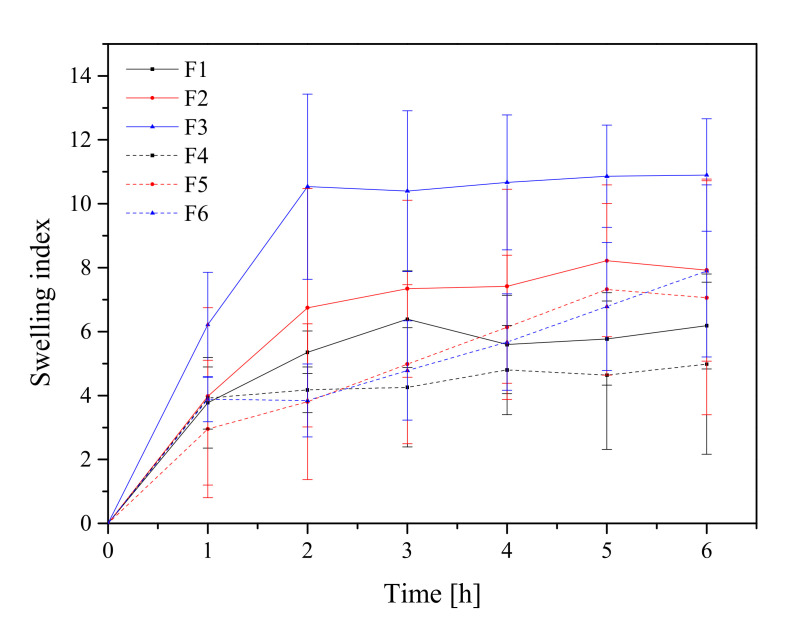
Swelling index study of formulations F1–F6.

Formulations F1–F6 were submitted to the swelling study in order to investigate tablet hydration capability. Adhesion occurs shortly after the beginning of swelling and increases with the degree of hydration of polymer. All formulations’ curves showed an initial rapid increase within the first hour and reached the highest value after 2 h for F1–F3 (Figure 5). Otherwise, formulations with higher polymer content (F5 and F6) acted differently, where an increase in the swelling index up to 8 h was observed. It is worth noting that after 6 h, the disintegration of the tablets into smaller particles was noticed, which could have caused such a rapid and significant increase in SI, so the results only up to 6 h are presented in Figure 5. The conclusion was that the use of these two formulations (F5 and F6) has to be limited to 6 h since after this period of time, the maintenance of the correct pharmaceutical form cannot be guaranteed. Additionally, the swelling index within formulation with different compression pressures was compared; there was no statistically significant difference.

HPMC-based tablets are classified as swelling-controlled systems and are controlled by the rate of penetration of media and erosion of the matrix. The combination of HPMC and Carbopol^®^ in a drug delivery system has been reported to improve the mucoadhesiveness. For instance, Marcos et al. studied the potential of combining Carbopol^®^ 974P and HPMC K4M using propranolol hydrochloride as a model drug and found that the amount of water imbibed in Carbopol^®^ and HPMC alone was lower than that of 1:1 mixture of two polymers [66]. The swelling behavior of the Carbopol^®^ is attributed to the uncharged –COOH group that is hydrated by forming hydrogen interactions with the imbibing water and, therefore, extending the polymer chain [66]. Importantly, the tablets formed at a compression pressure of 60 MPa met the conditions of the appropriate hardness, the swelling index was higher compared with the other tablets, and therefore those tablets were selected for further research.

**Figure 6:**
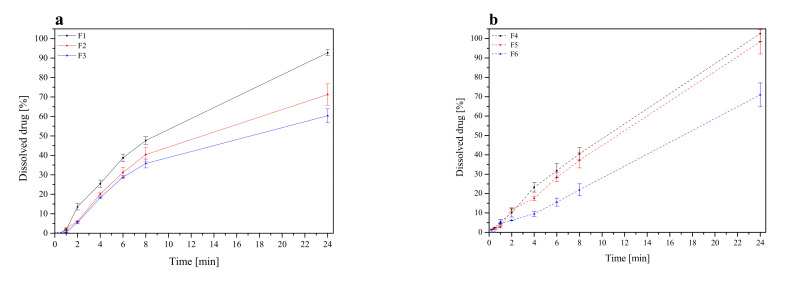
Resveratrol dissolution profiles from tablets F1–F3 (**a**) and F4–F6 (**b**).

The release profile of RSV from tablets containing lyophilized cyclodextrin-based *Polygoni cuspidati* extract is displayed (Figure 6). Profound differences in RSV release kinetics between formulations were noticed. Basically, a faster dissolution rate was observed for HPMC-based tablets (F1–F3). HPMC can be used in a controlled release drug delivery system due to its hydrophilic nature and fast hydration [66]. RSV release from HPMC matrices was controlled by the hydration of HPMC, which formed a gelatinous barrier layer at the surface of the matrix. It should be noted that an increase in HPMC concentration from 20% to 33% (formulation F1 and F4) was responsible for a decrease in RSV release in the first 8 h. In turn, formulations containing Carbopol^®^ exhibited a prolonged dissolution profile. Carbopol^®^ is a cross-linked polymer with high molecular weight and viscosity, and, when contacted with water, it swells and hold water inside its microgel network. As it was said, the RSV released slightly less from the tablets, the mucoadhesion matrix of which consisted of Carbopol^®^ and HPMC in the ratio 1:2, and the slowest from a matrix of Carbopol^®^ and HPMC in the ratio 1:1. At pH 6.8, the carboxylic groups in Carbopol^®^ were ionized and repelled one another, causing a maximum swelling, resulting in fewer and smaller regions of microviscosity [67]. The rapid gel formation acted as a barrier for the drug diffusion, thus prolonging its release. Moreover, a combination of anionic Carbopol^®^ and nonionic HPMC polymer produced synergistic inclusion in viscosity which could be due to H-interactions between carboxyl groups of Carbopol^®^ and OH groups of HPMC that led to stronger cross-linking between two polymers and retardation of the drug release [68].

On the basis of the above results, it was found that a combination of Carbopol^®^ and HPMC is more effective than only HPMC in sustaining the RSV release. Carbopol^®^ is a lightly crosslinked polymer, unlike cellulosic materials, which are linear. In the matrix of tablets prepared with linear hydrophilic polymers (such as HPMC), without a covalently crosslinked structure, a gelatinous layer is formed on the surface of the tablets during hydration. On the other hand, the crosslinked network of Carbopol^®^ enables the entrapment of the drug in the hydrogel domains. These hydrogels erode slower than what occurs in the case of linear polymers [67].

The mechanism of drug release from matrices containing swellable polymers is complex. To investigate what mechanism is responsible for the prolonged release, dissolution data obtained for the formulation F1–F6 were fitted to the following release models of zero-order, first-order equations, Higuchi model (applied for matrix systems), and Korsmeyer-Peppas model (employed for swellable matrices) (Supplementary Materials, Table S10). As the most probable, zero-order release was shown, which indicate that such a release is constant over a period of time. Additionally, a good fit to Korsmeyer-Peppas with ‘*n*’ values above 0.5 indicated the release approximates non-Fickian diffusion release mechanism. The relative complexity of the prepared formulae may indicate that the drug release is controlled by more than one mechanism; a coupling of diffusion and erosion. The results are in line with those conducted previously [67,69].

**Figure 7:**
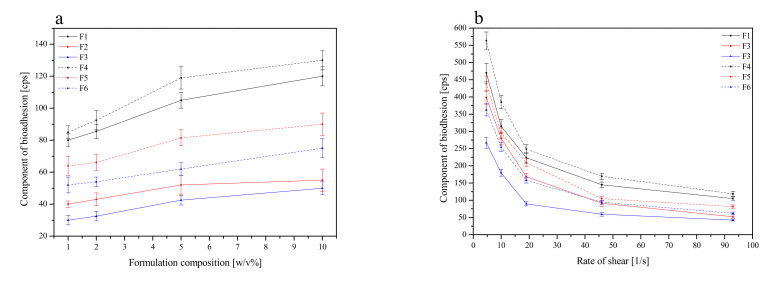
Effect of polymer concentration (1–10%) on the component of bioadhesion for formulations F1–F6 at rate of shear 93 1/s (**a**), and effect of the rate of shear on the component of bioadhesion for 5% of formulations F1–F6 (**b**).

The mucoadhesive properties of formulations F1–F6 were evaluated by rheological measurements. Based on those, the adhesive force of single polymer HPMC-blends was the strongest among the three types of blends (formulations F1 and F4), with a decreasing strength of adhesion observed with a decrease in the HPMC content and an increasing Carbopol^®^ content, which is in line with previous outcomes [67,70]. Therefore, all formulations could be compared as follows with the decreasing order of adhesion: blends containing HPMC > blends containing Carbopol^®^-HPMC 1:4 *m*/*m* > blends containing Carbopol^®^-HPMC 1:1 *m*/*m* (Figure 7). Comparing blends with the same qualitative but different quantitative compositions (F1 vs. F4, F2 vs. F5 and F3 vs. F6), obviously, those containing more polymers showed higher bioadhesion. In the case of HPMC, as a non-ionic polymer, its mucoadhesion did not depend on the pH of the medium; therefore, it adhered at any pH. It has a large number of hydroxyl groups, thanks to which it creates physical bonds (including hydrogen interactions) with mucus components, significantly affecting the strength of mucoadhesion [71,72]. Moreover, the higher viscosities of the HPMC formulation (F1 and F4) had higher adhesion forces, perhaps due to their elasticity, hydrogen bonding, molecular weight and cross-linking. Viscosity represents internal forces, while adhesion force is defined as the force required to detach the polymer from the surface [72]. Similarly, Carbopol^®^ can interact with the mucin molecule by physically entangling the chain followed by hydrogen bonding with sugar residues on the oligosaccharide chains resulting in the formation of an enhanced gel network so that the mucoadhesion remains adhesive for longer periods of time [69].

**Figure 8:**
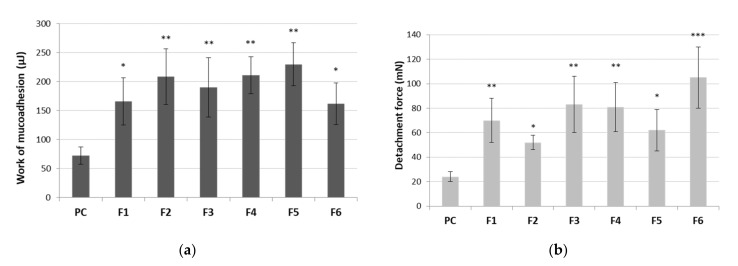
Work of mucoadhesion (**a**) and maximum detachment strength (**b**) of tablets containing cyclodextrin-based *Polygoni cuspidati* extract (F1–F6) (*n* = 5; mean ± SD). * represents significance with *p* ≤ 0.05, ** with *p* ≤ 0.01 and *** with *p* ≤ 0.001 in comparison to cellulose paper control (PC).

The average maximum detachment strength and work of mucoadhesion between tablets and the mucosal tissue are shown in Figure 8. The buccal mucosa from the porcine cheek was applied for simulation of buccal mucoadhesion due to its resemblance to human buccal mucosa in terms of anatomical structure and physiological activity [73].

All tablets showed statistically significant values of mucoadhesiveness as compared to the control (Figure 8). No alterations in work necessary to overcome the tablet–porcine buccal tissue interaction was observed between formulations (Figure 8a), which may point toward a relatively comparable separation manner of all tablets from the tissue. In contrast, some differences in detachment strength among tested formulations were noticed. Basically, an increase in the total content of polymer ingredients resulted in an increase in mucoadhesive strength of about 14% (F1 vs. F4), 16% (F2 vs. F5) and 20% (F3 vs. F6) (Figure 8b). The applied ratio of Carbopol^®^ to HPMC impacted the mucoadhesive strength of tablets. Surprisingly, the addition of Carbopol^®^ to HPMC in a ratio of 1:3 (formulations F2, F5) diminished HPMC’s ability to interact with mucosal tissue (Figure 8b). As the ratio of Carbopol^®^ to HPMC increased to 1:1 (tablets F3 and F6), an improvement in their maximum adhesive strength was observed, suggesting these formulations may be relatively more resilient to sharp tension (e.g., tongue movements shortly after application and wetting with saliva). Our observations are in agreement with data obtained by Kim et al. in which the bioadhesive force of buccal films altered significantly in respect to changes in polymers ratio in the formulation [74].

Tablets were additionally tested for their residence time to elaborate their mucoadhesive behavior upon continuous contact with the medium simulating saliva (Table 3). All examined formulations adhered immediately to the tissue swelled gradually in contact with the acceptor medium with no visible signs of disintegration throughout the test. Despite the continuous movement of the cylindrical probe, the contact time of tablets F2, F5, F6 with the mucosal surface was preserved within 180 min of test, whereas formulations F1, F3 and F4 separated from the tissue after 70, 80 and 30 min, respectively (Table 3). The presence of Carbopol^®^ appeared to improve the tablets’ retention upon hydration with simultaneous simulated buccal movements. The profoundly shorter retention time of formulation F4 most probably resulted from its excessive hydration within first 30 min of a study. The water absorption was responsible for an increase in weight which favored tablet separation from the mucosa.

## 4. Conclusions

The dried root and rhizome of *Polygonum cuspidatum* is a rich source of resveratrol, a well-known compound with strong antioxidant properties that inhibits the development of civilization diseases. As the amount of ethanol in the extraction mixture increases, the amount of extracted resveratrol and emodin increases. The addition of cyclodextrin at the stage of extraction caused a selective extraction of resveratrol from the plant material and resulted in an increase in the antioxidant and anti-inflammatory activity of lyophilized extracts (the best results were obtained for the L5 extract).

Presented studies displayed that tablets interaction and retention with the buccal tissue was improved when a combination of Carbopol^®^ 974 NF and HPMC polymers was used; however, modulating the ratio of Carbopol^®^ to HPMC in formulation influenced this behavior. The obtained results point toward different mucoadhesive behavior of tablets immediately after the application in a wetted or in swollen state upon the total hydration. The presence of Carbopol^®^ appeared to favor prolonged contact of tablets with mucosal tissue. Therefore, a matrix of tablets containing Carbopol^®^ and HPMC in ratio 1:1 (formulation F3) was found to compromise delivery systems’ resveratrol release and mucoadhesive properties.

## Figures and Tables

**Table 1:** Chemical composition of extracts and lyophilized extracts.

Extract Description	Extraction Mixture	Plant Raw Material/Extraction Modifier	Lyophilized Extract Description
W1	ethanol:water (3:7 *v*:*v*)	plant material	L1
W2	ethanol:water (3:7 *v*:*v*)	plant material + β-CD 1% *m*/*w*	L2
W3	ethanol:water (3:7 *v*:*v*)	plant material + β-CD 5% *m*/*w*	L3
W4	ethanol:water (5:5 *v*:*v*)	plant material	L4
W5	ethanol:water (5:5 *v*:*v*)	plant material + β-CD 1% *m*/*w*	L5
W6	ethanol:water (5:5 *v*:*v*)	plant material + β-CD 5% *m*/*w*	L6
W7	ethanol:water (5:5 *v*:*v*)	plant material + HP-β-CD 1% *m*/*w*	L7
W8	ethanol:water (5:5 *v*:*v*)	plant material + HP-β-CD 5% *m*/*w*	L8
W9	ethanol:water (7:3 *v*:*v*)	plant material	L9
W10	ethanol:water (7:3 *v*:*v*)	plant material + β-CD 1% *m*/*w*	L10
W11	ethanol:water (7:3 *v*:*v*)	plant material + β-CD 5% *m*/*w*	L11
W12	ethanol:water (10:0 *v*:*v*)	plant material	-
W13	ethanol:water (10:0 *v*:*v*)	plant material + β-CD 1% *m*/*w*	-
W14	ethanol:water (10:0 *v*:*v*)	plant material + β-CD 5% *m*/*w*	-

**Table 2:** Chemical composition of extracts and lyophilized extracts.

	F1	F2	F3	F4	F5	F6
	Content (mg) of compounds in one tablet					
*Polygoni cuspidati* lyophilized extract L5	80.0	80.0	80.0	80.0	80.0	80.0
Carbopol^®^ 974 NF	-	5.0	10.0	-	10.0	20.0
HPMC (15,000 cP)	20.0	15.0	10.0	40.0	30.0	20.0
Magnesium stearate	1.0	1.0	1.0	1.0	1.0	1.0
Sum	101.0	101.0	101.0	121.0	121.0	121.0

**Table 3:** Residence time (expressed in min) of tablets with *Polygoni cuspidati* extract (formulations F1–F6) to the porcine buccal mucosa (*n* = 3; mean).

Formulation	F1	F2	F3	F4	F5	F6
Residence time (min)	50	>180	80	30	>180	>180

## Data Availability

The data presented in this study are available through whole manuscript and supplementary material.

## Supplementary Materials

The following are available online at https://www.mdpi.com/article/10.3390/pharmaceutics13111916/s1, Table S1: Solid content of lyophilized extracts, Table S2: Validation parameters, Table S3: The content of resveratrol and emodin in prepared liquid and lyophilized extracts, Table S4: Apparent permeability value P_app_ for resveratrol and emodin, Table S5: The antioxidant effect of liquid extracts, Table S6: The antioxidant effect of lyophilized extracts, Table S7: Antioxidant activity of lyophilized extracts, Table S8: Effect on BChE activity, Table S9: Anti-inflammatory activity, Table S10: Mathematical characteristics of the resveratrol release kinetics from tablets F1–F6.
