# Supplementary material for: Cyclodextrin as Functional Carrier in Development of Mucoadhesive Tablets Containing Polygoni cuspidati Extract with Potential for Dental Applications
Source: Pharmaceutics. 2021 Nov 12;13(11):1916. doi: 10.3390/pharmaceutics13111916 (PMC8619530; doi:10.3390/pharmaceutics13111916)
Supplement: Supplementary file 1 [file pharmaceutics-13-01916-s001.zip › sup.pdf]

pharmaceutics

Supplementary Materials: Cyclodextrin as functional carrier in development of mucoadhesive tablets containing *Polygoni cuspidati* extract with potential for dental applications

Magdalena Paczkowska-Walendowska, Emilia Szymańska, Katarzyna Winnicka, Dominik Szwajgier, Ewa Baranowska-Wójcik, Marek A. Ruchała, Marek Simon, Judyta Cielecka-Piontek

Table S1. Solid content of lyophilized extracts

Lyophilized extract description	Solid content [%]
L1	8.87
L2	22.08
L3	38.44
L4	6.68
**L5**	21.01
L6	16.32
L7	27.12
L8	97.62
L9	2.12
L10	5.51
L11	4.33

Table S2. Validation parameters

Parameter	RSV	Emodin
Linearity: y = *a*x + *b*		
$a \pm S_a$	0.409 ± 0.009	0.297 ± 0.003
$b \pm S_b$	insignificant (α=0.05)	
Correlation coefficient (*r*)	0.999	0.999
Range of linearity [µg/mL]	25.0–500.0	25.0–500.0
Intra-day precision, RSD (<5% required) = repeatability		
0.4 [µg/mL]	0.354	0.838
4.0 [µg/mL]	0.972	1.659
40.0 [µg/mL]	0.579	2.226
Inter-day precision = reproducibility		
0.4 [µg/mL]	0.919	2.502
4.0 [µg/mL]	1.039	0.831
40.0 [µg/mL]	1.264	0.853
Limit of detection (LOD) [µg/mL]	31.84	16.70
Limit of quantification (LOQ) [µg/mL]	96.47	50.61

S_a standard deviation of slope; S_b standard deviation of intercept, *t*, calculated values of Student's t test, $t_{\alpha,f}$ = 2.228 critical values of Student's test for degrees of freedom f = 10 and significance level α = 0.05.

Table S3. The content of resveratrol and emodin in prepared liquid and lyophilized extracts

Liquid extract			Lyophilized extract		
extract description	Content ± SD [µg per 1g of plant material]		lyophilized extract description	Content ± SD [µg per 1mg of lyophilized extract]	
	RSV	emodin		RSV	emodin
W1	44.42 ± 14.26	0.56 ± 0.14	L1	3.52 ± 0.52	0.07 ± 0.01
W2	279.60 ± 13.33	0.63 ± 0.58	L2	1.08 ± 0.15	0.02 ± 0.01
W3	200.93 ± 25.93	0.20 ± 0.08	L3	0.59 ± 0.11	0.03 ± 0.01
W4	13.25 ± 2.52	3.84 ± 0.37	L4	3.61 ± 0.23	4.38 ± 0.48
**W5**	**297.40 ± 26.27**	**3.42 ± 0.27**	**L5**	**1.87 ± 0.24**	**1.91 ± 0.20**
W6	196.19 ± 23.37	1.95 ± 0.20	L6	1.67 ± 0.24	2.74 ± 0.49
W7	476.92 ± 52.80	36.79 ± 2.73	L7	1.27 ± 0.09	1.92 ± 0.12
W8	399.62 ± 24.42	141.57 ± 24.56	L8	0.36 ± 0.01	1.17 ± 0.43
W9	66.09 ± 12.24	5.36 ± 0.89	L9	2.25 ± 0.98	5.72 ± 2.55
W10	434.60 ± 42.13	44.47 ± 23.28	L10	3.26 ± 0.37	11.12 ± 1.48
W11	280.57 ± 25.58	32.01 ± 6.79	L11	2.18 ± 0.07	10.55 ± 1.62
W12	401.18 ± 5.06	353.88 ± 11.99	-	-	-
W13	446.11 ± 5.60	364.37 ± 16.06	-	-	-
W14	402.23 ± 4.13	342.19 ± 12.62	-	-	-

Table S4. Apparent permeability value P_{app} for resveratrol and emodin

Liquid extract			Lyophilized extract		
extract description	P_{app} ± SD x 10^{-6} [cm/s]		lyophilized extract description	P_{app} ± SD x 10^{-6} [cm/s]	
	RSV	emodin		RSV	emodin
standards	7.34 ± 0.28	7.42 ± 0.97	standards	7.34 ± 0.28	7.42 ± 0.97
W1	15.71 ± 1.83	13.19 ± 0.55	L1	55.08 ± 0.45	52.28 ± 0.49
W2	32.93 ± 3.81	13.47 ± 3.16	L2	49.32 ± 5.70	47.87 ± 1.09
W3	63.73 ± 2.27	9.06 ± 2.81	L3	66.03 ± 5.42	50.68 ± 4.23
W4	55.00 ± 5.54	8.03 ±1.20	L4	62.41 ± 3.49	41.06 ± 3.39
**W5**	**44.03 ± 5.73**	**7.62 ± 3.17**	**L5**	**55.84 ± 0.60**	**38.19 ± 2.98**
W6	48.05 ± 2.46	8.70 ± 1.55	L6	50.56 ± 1.10	44.46 ± 3.87
W7	62.18 ± 5.86	13.60 ± 0.24	L7	59.38 ± 5.44	41.55 ± 3.45
W8	57.59 ± 4.33	18.44 ± 1.02	L8	59.09 ± 1.16	53.98 ± 1.22
W9	56.81 ± 3.84	8.97 ± 0.83	L9	45.06 ± 6.19	45.75 ± 3.31
W10	53.33 ± 5.93	7.01 ± 0.61	L10	44.60 ± 6.33	29.29 ± 1.26
W11	73.96 ± 1.80	8.51 ± 0.54	L11	49.02 ± 1.54	18.61 ± 2.12
W12	93.27 ± 3.54	7.01 ± 0.89	-	-	-
W13	80.03 ± 3.29	8.31 ± 0.95	-	-	-
W14	70.19 ± 4.60	8.61 ± 0.97	-	-	-

Table S5. The antioxidant effect of liquid extracts

Liquid extract description	polyphenolic compounds content [GAE mg/1,0 g material]	DPPH IC_{50} [µg/mL]	CUPRAC $IC_{0.5}$ [µg/mL]
W1	44.6	0.19	0.22
W2	46.1	0.28	0.32
W3	49.3	0.25	0.33
W4	40.3	0.22	0.29
**W5**	**54.5**	**0.16**	**0.27**
W6	53.3	0.26	0.31
W7	84.1	0.24	0.28
W8	70.5	0.22	0.26
W9	38.4	0.21	0.25
W10	52.9	0.16	0.23
W11	49.8	0.27	0.30
W12	38.3	0.38	0.39
W13	33.6	0.29	0.43
W14	33.7	0.42	0.45
RSV		0.0257	0.032
Vitamin C		0.0097	0.010
emodin		>4.0	>4.0

Table S6. The antioxidant effect of lyophilized extracts

Lyophilized extract description	DPPH IC_{50} [µg/mL]	CUPRAC $IC_{0.5}$ [µg/mL]	FRAP Equivalent Trolox concentration [mg/mL]	HORAC Equivalent gallic acid concentration [µg/cm³]	ORAC Equivalent Trolox concentration [µM/L]
L1	33.1	12.9	0.18 ± 0.01	351.1 ± 11.2	29.4 ± 1.2
L2	73.1	73.2	0.18 ± 0.02	**482.3 ± 18.3**	28.6 ± 3.1
L3	145.1	137.1	0.19 ± 0.02	475.0 ± 10.3	30.5 ± 2.4
L4	35.1	4.0	0.20 ± 0.01	453.3 ± 8.2	31.3 ± 2.0
**L5**	**66.1**	**59.6**	**0.32 ± 0.03**	**481.3 ± 20.3**	35.6 ± 1.1
L6	83.1	78.5	0.23 ± 0.01	476.3 ± 21.0	**39.9 ± 1.9**
L7	100.1	96.8	0.19 ± 0.04	476.5 ± 17.3	27.6 ± 2.6
L8	190.1	309.9	0.16 ± 0.03	**490.0 ± 26.3**	26.4 ± 0.8
L9	36.1	33.8	0.17 ± 0.10	431.6 ± 20.4	23.4 ± 2.2
L10	44.1	53.3	0.20 ± 0.01	458.2 ± 16.3	25.0 ± 1.4
L11	46.1	49.7	0.24 ± 0.02	463.4 ± 28.3	**42.4 ± 1.0**
RSV	0.0257	0.032			

Vitamin C	0.0097	0.010
emodin	>4.0	>4.0

Table S7. Antioxidant activity of lyophilized extracts

	Effect on SOD activity	Effect on GR and GPx activity				Linoleic acid oxidation	β-carotene oxidation
Lyophilized extract description	Enzyme inhibition [%]	GR inhibition (%)	GR inhibitory activity (μmol consumed NADPH/min incubation)	GPx inhibition (%)	GPx inhibitory activity (nmol consumed NADPH/min incubation)	Equivalent ascorbic acid concentration (mg/ml)	Equivalent ascorbic acid concentration (μg/ml)
L1	23.6 ± 2.5	56.8 ± 1.3	2.2 ± 0.2	**66.2 ± 2.3**	132.5 ± 4.6	1.34 ± 0.15	27.0 ± 3.2
L2	24.8 ± 3.1	58.0 ± 3.2	2.2 ± 0.2	46.6 ± 1.5	92.9 ± 3.5	1.64 ± 0.21	29.6 ± 2.1
L3	20.5 ± 2.0	60.1 ± 2.6	2.3 ± 0.1	53.3 ± 2.5	106.3 ± 4.2	1.83 ± 0.04	28.4 ± 0.3
L4	21.4 ± 1.9	67.3 ± 3.0	2.6 ± 0.1	53.1 ± 4.6	105.9 ± 6.7	2.05 ± 0.19	34.2 ± 3.3
**L5**	**28.7 ± 3.0**	**79.0 ± 2.8**	3.1 ± 0.2	**60.0 ± 5.2**	119.6 ± 7.0	2.56 ± 0.11	36.7 ± 4.2
L6	**31.4 ± 4.0**	**83.3 ± 2.5**	3.2 ± 0.2	31.4 ± 3.2	62.6 ± 4.3	2.01 ± 0.10	38.4 ± 1.3
L7	23.2 ± 2.2	65.7 ± 3.2	2.6 ± 0.3	16.9 ± 5.1	33.7 ± 7.0	2.16 ± 0.09	27.1 ± 2.6
L8	20.2 ± 3.3	62.4 ± 1.8	2.4 ± 0.3	16.7 ± 4.2	33.4 ± 5.6	1.99 ± 0.13	20.6 ± 0.9
L9	19.6 ± 3.9	65.4 ± 1.9	2.6 ± 0.2	25.4 ± 3.0	50.6 ± 4.9	1.84 ± 0.25	28.5 ± 2.2
L10	27.5 ± 1.7	76.5 ± 2.2	3.0 ± 0.1	46.6 ± 2.4	92.9 ± 3.2	2.21 ± 0.21	30.2 ± 2.0
L11	**32.6 ± 2.3**	**78.4 ± 2.9**	3.1 ± 0.1	25.4 ± 3.1	50.6 ± 3.6	2.11 ± 0.06	35.4 ± 1.6

Data expressed as mean ± SD; The best values in bold

Table S8. Effect on BChE activity

**Sample**	**Equivalent reference concentration [μg/mL]**				
	**neostigmine**	**magniflorine**	**donepezil**	**eserine**	**rivastigmine**
L1	3.6 ± 0.1	11.4 ± 0.1	2.1 ± 0.1	2.4 ± 0.1	18.7 ± 0.1
L2	3.9 ± 0.1	12.6 ± 0.1	2.3 ± 0.1	2.6 ± 0.1	20.5 ± 0.0
L3	1.4 ± 0.1	4.6 ± 0.1	0.8 ± 0.0	0.9 ± 0.0	7.5 ± 0.0
L4	4.3 ± 0.1	13.7 ± 0.0	2.5 ± 0.1	2.8 ± 0.0	22.4 ± 0.1
L5	3.2 ± 0.0	10.3 ± 0.1	1.9 ± 0.0	2.1 ± 0.1	16.8 ± 0.0
L6	3.6 ± 0.1	11.4 ± 0.0	2.1 ± 0.1	2.4 ± 0.0	18.7 ± 0.0
L7	1.4 ± 0.0	4.6 ± 0.0	0.8 ± 0.1	0.9 ± 0.1	7.5 ± 0.0
L8	1.1 ± 0.0	3.5 ± 0.1	0.6 ± 0.1	0.7 ± 0.0	5.6 ± 0.1
L9	2.9 ± 0.0	9.1 ± 0.1	1.7 ± 0.0	1.9 ± 0.0	14.9 ± 0.1
L10	1.8 ± 0.1	5.7 ± 0.0	1.0 ± 0.1	1.2 ± 0.2	9.3 ± 0.2
L11	2.2 ± 0.1	6.9 ± 0.0	1.2 ± 0.1	1.4 ± 0.1	11.2 ± 0.1

Table S9. Anti-inflammatory activity

**Sample**	**Equivalent acetylsalicylic acid concentration (mg/cm³)**	**COX-2 inhibition [%]**
L1	3.26 ± 0.2	92.3 ± 4.3
L2	3.24 ± 0.0	87.2 ± 3.2
L3	3.15 ± 0.1	64.1 ± 2.4
L4	3.27 ± 0.1	**94.9 ± 2.1**
L5	3.24 ± 0.1	87.2 ± 1.6
L6	3.29 ± 0.0	**96.0 ± 4.2**
L7	2.99 ± 0.0	74.4 ± 3.3
L8	3.00 ± 0.0	71.8 ± 2.0
L9	3.24 ± 0.1	87.2 ± 3.5
L10	3.26 ± 0.1	**92.3 ± 4.1**
L11	3.24 ± 0.1	87.2 ± 3.2

Table S10. Mathematical characteristics of the resveratrol release kinetics from tablets F1-F6

Formulation	Mathematical model								
	Zero-order kinetic		First-order kinetic		Higuchi kinetic		Korsmeyer-Peppas kinetic		
	K	R^2	K	R^2	K	R^2	K	R^2	n
F1	3.99	**0.943**	0.187	0.563	8.79	0.761	1.42	0.895	0.593
F2	3.11	**0.922**	0.177	0.599	6.93	0.764	1.33	0.921	0.554
F3	2.66	**0.899**	0.183	0.566	6.05	0.775	1.49	0.811	0.544
F4	4.29	**0.992**	0.169	0.635	8.64	0.675	0.99	0.978	0.548
F5	4.13	**0.995**	0.178	0.642	8.14	0.651	1.19	0.949	0.554
F6	2.95	**0.997**	0.167	0.700	5.46	0.578	1.12	0.926	0.489
